# Determinants of China’s development assistance for health at the sub-national level of African countries (2006–2015)

**DOI:** 10.1186/s40249-018-0510-8

**Published:** 2018-12-19

**Authors:** Hao-min Yang, Pei-long Liu, Yan Guo

**Affiliations:** 1 Department of Medical Epidemiology and Biostatistics, Karolinska Institutet, Stockholm, Sweden; 2 Department of Global Health, School of Public Health, Peking University, Beijing, China; 3 Department of Health Policy and Management, School of Public Health, Peking University, Xueyuan Road 38, Haidian District, Beijing, 100191 China

**Keywords:** China, Africa, Development assistance for health

## Abstract

**Background:**

Despite the increasing interest in China’s development assistance for health (DAH) in African countries, little is known regarding the distribution and determinants of China’s DAH project allocation among the principle subdivisions (provinces & states) within African countries.

**Methods:**

We mapped the distribution of China’s DAH projects in 670 principle subdivisions of 50 African countries during 2006–2015 using web-based information. The political, demographic, health and socioeconomic indicators of DAH allocation were analyzed using conditional logistic regression models. The national capital city and political leader’s birth place were selected as the main political indicators, and health indicators were selected according to different fields of the DAH projects.

**Results:**

China’s DAH projects (mainly China medical teams [CMTs], hospitals and anti-malaria centers) were mostly allocated to the western and eastern coasts of Africa, although CMTs were also dispatched to northern Africa. National capital cities were significantly associated with the allocation of China’s DAH projects (*P* <  0.001). Anti-malaria centers were more likely to be allocated to principle subdivisions with larger populations (*OR* = 1.35), and CMTs were allocated to subdivisions with high population densities (*OR* = 79.01). No health-related indicators were identified to affect project allocation except for the facility delivery rate and under-five mortality rate, which were associated with hospital allocation. We also found an association between CMT allocation and the use of artemisinin-based combination therapy in children.

**Conclusions:**

Allocation of China’s DAH projects is strongly affected by political and demographic factors. Implementation of China’s new DAH projects should target health and socio-economic indicators and impact metrics in scaling up tailored and cost-effective programs in Africa.

**Electronic supplementary material:**

The online version of this article (10.1186/s40249-018-0510-8) contains supplementary material, which is available to authorized users.

## Multilingual abstracts

Please see Additional file 1 for translations of the abstract into the five official working languages of the United Nations.

## Background

China has provided development assistance for health (DAH) to African countries for more than 60 years, even when its per capita gross domestic product (GDP) was fairly low. With greater economic strength and a rising international status, China’s DAH has experienced a booming period after initiation of the Beijing Declaration in 2006 [1]. In 2015, China proposed a series of new DAH programs, including 100 maternal and child health projects, 100 hospitals & clinics, and the China-Africa cooperation plan for public health [2–5]. How China will allocate these DAH projects and consequently contribute to the realization of health-related Sustainable Development Goals in other developing countries has attracted global attention [6].

Since the decision-making department of China’s development assistant projects was involved in the Ministry of Commerce, several studies had already tested commercial and political determinants for the allocation of China’s DAH projects at the national level. They identified political relationship with China to be the major determinants for DAH [7], while no association was found between DAH projects and import/export trade [8–10].

However, little is known about the allocation of China’s DAH projects at the subnational level. For China’s development assistance overall, African leaders’ birth places, national capital cities and economic development levels are important indicators for aid allocation at the subnational level [11]. Compared to overall development assistance, studies on the political and demographic determinants of China’s DAH allocation are scarce at the subnational level [9].

Both China’s and African countries’ health departments are involved in the projects during the implementation stage. Therefore, these departments may influence project allocation within African countries. Since DAH is also an approach to transfer donors’ experiences in health development to the recipient country [12], DAH projects may be allocated to provinces where health indicators are worse and the specific experience is needed. China has obtained great achievements in disease prevention, maternal and child health, and health system strengthening during the previous decades [13–15]. However, whether the corresponding local health indicators in African countries have influenced China’s DAH project allocation is unknown.

To better understand the subnational allocation of China’s DAH projects during this booming period (2006–2015) and to provide evidence for improved allocation of new projects, we attempted to describe the distribution of China’s main DAH projects in this study. We also aimed to analyze the political, demographic, health and socioeconomic determinants for subnational allocation of DAH projects.

## Methods

### Data source

In this study, principle subdivision (e.g., province or state) of a country was considered as a subnational entity and also the object of study. Two datasets were used in this study: 1) principle subdivisions of all African countries with diplomatic relationships with China and 2) a subset of the countries that conducted phase five Demographic and Health Surveys (DHSs).

During 2006–2015, 670 principal subdivisions in 50 African countries were involved in this study, including Malawi, Chad and South Sudan, which established or reestablished diplomatic relationships with China during this period (Table 1). Four African countries (Swaziland, Sao Tome and Principe, Gambia and Burkina Faso) were excluded from the study because they had no diplomatic relationship with China during this period and therefore could not receive China’s DAH. We coded the principal subdivisions according to the International Organization for Standardization (ISO-3166-2) [16]. The ISO-3166-2 code consists of two parts linked by a hyphen: the first part consists of two alphanumeric codes that represent the country, and the second part with less than 3 characters represents the principal subdivisions in the country. The outcomes for this study were different forms of DAH projects from China, and the explanatory variables included several demographic, political, economic, health and social indicators.

**Table 1:** Descriptive of the two datasets analyzed for allocation of China’s development assistance for health in Africa

	All available countries	Subsets of countries with DHSs
Number of countries	50	23
Eastern Africa	18	10
Middle Africa	8	2
Northern Africa	6	1
Southern Africa	4	2
Western Africa	14	8
Number of principle subdivisions	670	211
Eastern Africa	193	92
Middle Africa	106	21
Northern Africa	152	7
Southern Africa	41	23
Western Africa	178	68
Number of CMTs, Total	82	39
Eastern Africa	27	18
Middle Africa	11	3
Northern Africa	22	6
Southern Africa	3	2
Western Africa	19	10
Number of hospitals, Total	35	17
Eastern Africa	14	8
Middle Africa	4	1
Northern Africa	1	0
Southern Africa	1	1
Western Africa	15	7
Number of anti-malaria centers, Total	30	17
Eastern Africa	10	9
Middle Africa	7	1
Northern Africa	1	0
Southern Africa	0	0
Western Africa	12	7

Abbreviations: *DHSs* Demographic and Health Surveys, *CMTs* China medical teams, There are altogether 54 countries in Africa by 2015, and four of them (Swaziland, Sao Tome and Principe, Gambia and Burkina Faso) were excluded in the study because they had no diplomatic relationship with China during this period and therefore would not receive China’s DAH. In these 50 countries, 23 of them conducted DHS and reported the health and social status indicators during 2003–2007. Regions of Africa were divided according to the United Nations Country Grouping

### Forms of DAH as the study target

China’s DAH projects during this period included dispatching China medical teams (CMTs), constructing hospitals, anti-malaria centers, training health professionals, donating health equipment and medicines, and the “Brightness Action” campaign. In this study, we focused on regular CMTs, hospitals and anti-malaria centers, which covered about 96% of China’s DAH to Africa during 2007–2011 and obtained continuous funding thereafter [17]. The decision making mechanism for these projects is listed in Additional file 2: Figure S1. Details of the different forms of DAH and information for each individual project were described in our previous publication [9]. Briefly, we searched data from multiple Chinese sources, including the ministries of health [18], commerce [19], and foreign affairs [20], provincial governments, and the official media press [21, 22] from the 1950s until 2011. In this study, we added those projects implemented during 2012–2015 using the same approach. We also searched and added some hospital projects with English sources documented in the AidData dataset [23] to obtain a comprehensive dataset of China’s DAH projects during 2006–2015. In addition, one officer from International Health Exchange and Cooperation Center, National Health and Family Planning Commission (one of the major implementation entities for China’s DAH) was asked to check the validity of our dataset. We added the ISO-3166-2 code for each project to identify its location (Additional file 2). Some medical teams were dispatched to different principal subdivisions of the country, and we coded accordingly. We also used the number of medical team members in each principal subdivision to show the project magnitude.

### Demographic, political and economic factors

Because the main targets of DAH projects are the African people, total population and population density were considered as the most important demographic factors. Therefore, we obtained the area and population of principal subdivisions from the national bureaus of statistics in all African countries [24]. For each principal subdivision, we imputed the population in each year based on statistics from the survey years with a linear regression model. In this study, we used the average population during 2003–2007 (the same reference period used for the health and social-related factors to maintain concordance) and calculated the population density in each principal subdivision.

National capital city and political leaders’ birth place were considered as the main indicators for political importance of the principle subdivisions and were shown to be associated with subnational allocation of China’s development assistance projects overall [11]. Here, we wanted to test whether these two factors also influenced the allocation of DAH projects. We used an updated version of the Archigos dataset of countries’ effective leaders [11, 25], and linked the leaders’ birth places into our dataset.

GDP is one important indicator for economic development and welfare and may influence the allocation of DAH projects. However, GDP is often measured poorly in developing countries, especially at the subnational level, considering the weak performance of government statistic departments [26]. To measure economic development at the subnational level, we used the nighttime light intensity, which is an established proxy for GDP at both the national and subnational levels [26, 27]. The National Oceanic and Atmospheric Administration provides pixel data on nighttime light annually with a resolution of approximately one square kilometer that can be used to calculate the average nighttime light intensity at the subnational level. In this analysis, we used pixel data for the year 2007.

### Health and social related factors

In the subset study, we selected 23 African countries that had conducted phase five Demographic and Health Surveys supported by the United States Agency for International Development during 2003–2007 (Table 1). The DHSs were initiated in 1984 to collect comparable national household data and monitor vital statistics and population health indicators in low- and middle-income countries [28]. The DHSs were usually conducted every 5 years, and we chose this period to avoid the reverse causality problem. Summary statistics for each principal subdivision were directly used for the analysis. The DHS principal subdivisions in Morocco, Liberia and Nigeria were different from the ISO-3166-2 subdivisions, and we therefore recoded these principal subdivisions using temporary codes with the same structure as the ISO-3166-2 code and only used them in analysis of the DHS-related data.

Since China has made remarkable progress in strengthening their health system, some of China’s DAH projects may be used to improve the African people’s accessibility to health care (e.g., enhancing the quality and quantity of local health personnel and building health facilities for the local people) [17]. Therefore, we selected the following indicators to evaluate this potential determinant of China’s DAH allocation: percentage of the population with financial problems for health care accessibility, percentage of the population with distance problems for health care accessibility and percentage of the population with transport problems for health care accessibility.

Another highlighted field in which China has achieved great success is maternal and child health, which is still a challenge in African countries. Hence, we also selected some maternal and child health indicators, such as health facility-based delivery rate (per 1000 live births), under five mortality rate (per 1000 live births) and low birth weight rate in the previous five years. Some indicators for malaria prevention and treatment were also involved in the analysis, because anti-malaria campaign was one of China’s main DAH approaches during this period. These indicators included the percentage of households with access to insecticide-treated mosquito net (ITN) and the percentage of children treated with artemisinin-based combination therapy (ACT) when they had a fever in the previous two weeks. We also obtained some social-related factors for the principal subdivisions, such as the percentages of male and female literacy and the male and female unemployment rates.

### Statistical analysis

To show the geographic distribution of China’s DAH projects, we linked our data of China’s DAH projects to the Database of Global Administrative Areas [29] by use of the ISO-3166-2 code. These projects were mapped using ArcGIS 10.2 (ESRI, CA, USA).

To examine the statistical association between the subnational allocation of China’s DAH projects and the demographic, political and economic indicators of the principal subdivisions, we used a conditional logistic regression model to calculate the odds ratios for each outcome (allocation of the CMTs, hospitals or anti-malaria centers). All three outcomes were coded as a binary variable indicating whether the principal subdivision had this form of DAH project. The conditional logistic regression model was conditioned on the country’s fixed effect of aid allocation, including several confounders (e.g., the governance capacity and development stage) that might influence the tested association and were sometimes difficult to measure. Since these confounders were not the focus of this study, conditional logistic regression model was an efficient approach to control these confounders. Two models were performed to estimate the odds ratios: a univariate model that only included one potential factor and a multivariate model that included all five indicators (the leader’s birth place, national capital city, average population, population density and nighttime light) in the model. We also tested the potential collinearity between average population and population density, which had an acceptable variance inflation factor (VIF) of less than 1.5.

The associations between health and social-related factors and China’s DAH allocation were also tested using the same approach. The only exception was that the multivariate model in this analysis was adjusted by the five political, demographic and economic indicators, because these indicators were considered as confounders for potential associations between the allocation of China’s DAH and health and social indicators of the principle subdivisions.

The statistical analyses were performed using SAS 9.4 (SAS Institute Inc., Cary, NC, USA) and Stata 14.0 software (Stata Corporation, College Station, TX, USA).

## Results

### Geographic distribution of DAH projects

**Fig. 1:**
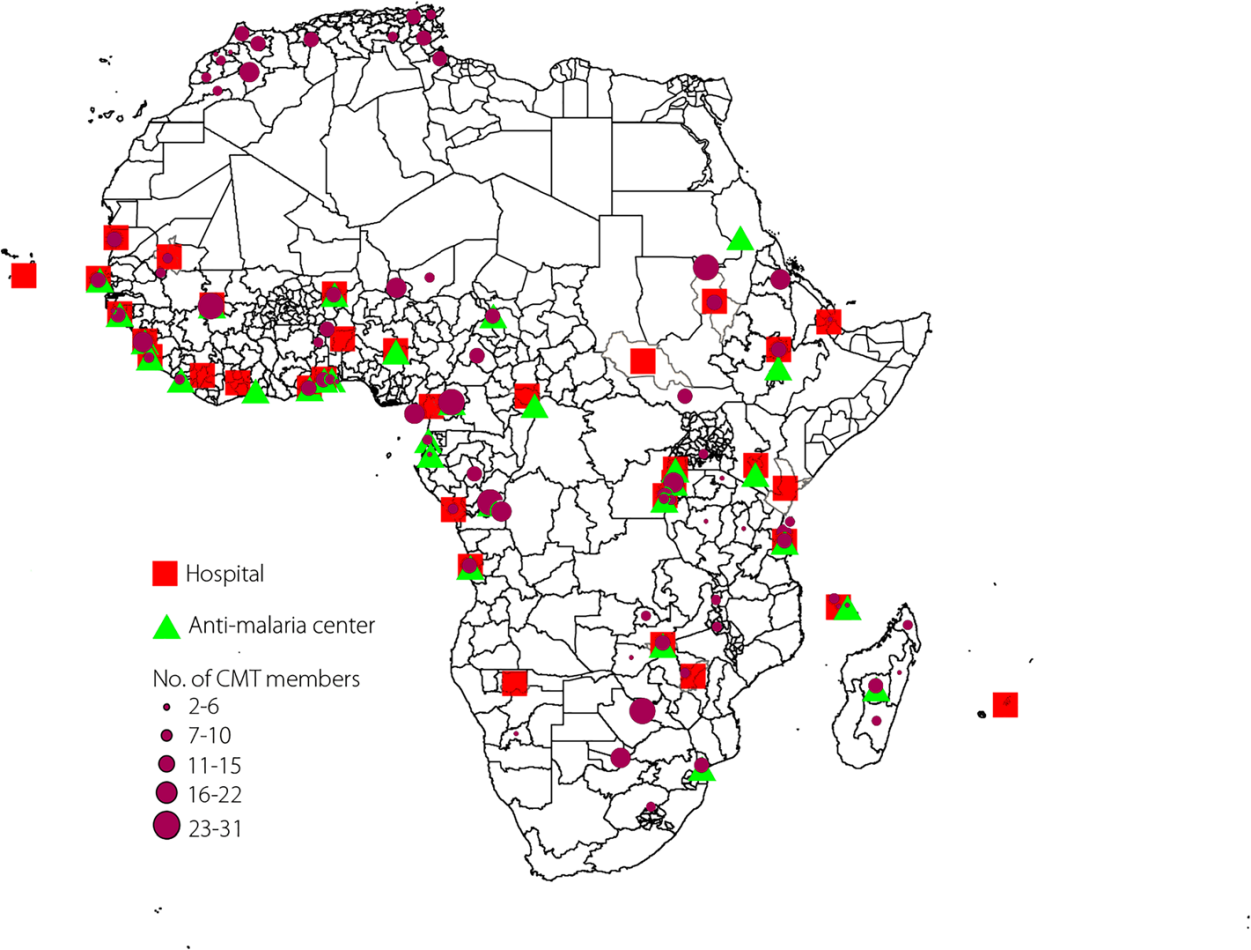
Sub-national distribution of China’s development assistance for health in Africa. Shapes of the principal subdivisions and their boundaries were provided by the Database of Global Administrative Areas

The geographic distribution of China’s DAH projects at the subnational level is shown in Fig. 1. Three clusters of DAH allocation were found (northern Africa along the Mediterranean Sea and along the western and eastern coasts of the African continent). No DAH project was allocated to the Saharan area and the central inland of Africa. Only medical teams were allocated to the northern African coast. Majority of the principal subdivisions allocated with medical teams had 11–22 team members. More than 80% of the hospitals and 70% of the anti-malaria centers were allocated to the western and eastern regions of Africa (Table 1). No country from southern African received aid for an anti-malaria center, and only Sudan from northern Africa had this type of DAH.

### Determinants of CMT allocation

The univariate analyses identified many political, demographic, socioeconomic and health indicators for the subnational allocation of CMTs, although the effects of health and social indicators were attenuated after multivariate adjustment. Leader’s birth place was only associated with CMT allocation in the univariate model, with a crude odds ratio (c*OR*) of 1.97 (*P* = 0.05). In the multivariate model, national capital cities, population numbers, population density and nighttime light intensity were all significantly associated with subnational allocation of CMTs (Table 2), with the nighttime light intensity inversely associated with CMT allocation (adjusted odds ratio [a*OR*] = 0.80, *P* = 0.03). The only health indicator associated with CMT allocation was a higher percentage of ACT use in children with a fever (a*OR* = 1.25, *P* = 0.018).

**Table 2:** Association between China’s development assistance for health allocation and subnational political and demographic characteristics

	Medical teams				Hospitals				Anti-malaria centers			
	Univariate model		Multivariate model		Univariate model		Multivariate model		Univariate model		Multivariate model	
	c*OR*	*P*-value	a*OR*	*P*-value	c*OR*	*P*-value	a*OR*	*P*-value	c*OR*	*P*-value	a*OR*	*P*-value
Political indicators												
Birth places of national leaders*	1.97	0.050	1.17	0.778	2.18	0.067	1.61	0.362	1.83	0.182	1.16	0.885
Capital city of the country	76.80	< 0.001	29.62	< 0.001	9.16	< 0.001	7.98	< 0.001	49.65	< 0.001	35.42	< 0.001
Demographic and economic indicators												
Number of population (per million increase)	1.27	0.090	2.02	0.004	1.02	0.848	0.92	0.638	1.90	0.001	1.35	0.015
Population density (1000/km^2^ increase)	40.30	< 0.001	79.01	0.006	1.27	0.014	0.84	0.324	1.99	< 0.001	1.63	0.382
Nighttime light (per scale increase)	1.22	< 0.001	0.80	0.031	1.10	< 0.001	1.06	0.147	1.16	< 0.001	0.96	0.707

Abbreviations: *DAH* Development Assistance for Health, *cOR* crude odds ratio, *aOR* adjusted odds ratio. Conditional logistic regression model was used to calculate the odds ratio for each outcome (allocation of the China medical teams, hospitals or anti-malaria centers). Univariate model only included one potential factor and multivariate model included all the five indicators. *P*-value was reported for both models

*Missingness on leaders’ birth place was 16.0% (*n* = 107 principle subdivisions in 5 countries)

### Determinants of hospital allocation

Although national capital city, population density and nighttime light intensity were associated with the subnational allocation of hospitals in the univariate model, national capital city was the only significant political indicator identified in the multivariate model, with an a*OR* of 7.98 (*P* <  0.001). Hospital allocation was not associated with health facility accessibility in the principle subdivisions. Interestingly, subdivisions with high facility delivery rates (*OR* = 1.08, *P* = 0.01) and low under-five mortality rates (*OR* = 0.97, *P* = 0.04) had the highest probability of allocation with hospital projects. We also found that a high female literacy rate was associated with subnational allocation of hospitals (a*OR* = 1.12, *P* = 0.02).

### Determinants of anti-malaria center allocation

Malaria was the main disease focus of China’s DAH during this period. However, none of the health-related indicators was associated with allocation of anti-malaria centers, even those indicators related to the malaria risk, such as access to ITN and ACT (Table 3). Instead, national capital city was significantly associated with the allocation of anti-malaria centers, with a strong a*OR* of 35.42 (*P* <  0.001). The anti-malaria centers were also more likely to be allocated to subdivisions with large population sizes (a*OR* = 1.35, *P* = 0.02).

**Table 3:** Association between allocation of China’s development assistance for health projects and sub-national health and social related factors

	Medical teams				Hospitals				Anti-malaria centers			
	Univariate model		Multivariate model		Univariate model		Multivariate model		Univariate model		Multivariate model	
	c*OR*	*P*-value	a*OR*	*P*-value	c*OR*	*P*-value	a*OR*	*P*-value	c*OR*	*P*-value	a*OR*	*P*-value
Causes of health facility accessibility problems												
Distance (% of the population)	0.91	< 0.001	0.96	0.214	0.89	< 0.001	0.93	0.201	0.89	< 0.001	1.01	0.705
Finance (% of the population)	0.93	< 0.001	1.01	0.717	0.88	< 0.001	0.92	0.262	0.92	0.002	1.03	0.294
Transport (% of the population)	0.90	< 0.001	0.95	0.106	0.91	< 0.001	0.94	0.242	0.91	< 0.001	1.02	0.468
Maternal and child health												
Facility delivery rate (% of live births in the previous 5 years)	1.06	< 0.001	1.01	0.614	1.08	< 0.001	1.08	0.015	1.09	< 0.001	0.99	0.754
Under 5 mortality rate(per thousand live births in previous 5 years)	0.98	< 0.001	1.00	0.927	0.97	0.002	0.97	0.040	0.96	< 0.001	0.99	0.521
Low birth weight rate (% of live births in previous 5 years)	1.00	0.966	1.05	0.367	0.93	0.270	0.93	0.452	1.01	0.785	1.10	0.075
Malaria prevention and treatment												
Access to an insecticide-treated mosquito net (% of the population)	1.03	0.302	0.90	0.218	1.05	0.138	1.02	0.661	1.08	0.059	1.02	0.494
ACT used in treatment(% of child with fever)	1.10	0.043	1.25	0.018	0.97	0.662	0.95	0.612	1.00	0.992	0.98	0.600
Social factors												
Male literacy rate (%)	1.12	< 0.001	1.04	0.268	1.11	< 0.001	1.08	0.092	1.12	< 0.001	0.99	0.748
Female literacy rate (%)	1.10	< 0.001	1.03	0.270	1.11	< 0.001	1.12	0.022	1.10	< 0.001	0.99	0.519
Male unemployment rate (%)	1.04	0.120	0.97	0.526	1.02	0.348	1.00	0.901	1.05	0.112	0.96	0.377
Female unemployment rate (%)	1.03	0.061	1.01	0.786	1.02	0.284	1.01	0.771	1.05	0.009	0.99	0.582

Abbreviations: *DAH* Development Assistance for Health; c*OR* = crude odds ratio; a*OR* = adjusted odds ratio; ACT: Artemisinin-based combination therapy. Conditional logistic regression model was used to calculate the odds ratio for each outcome (allocation of the China medical teams, hospitals or anti-malaria centers). Univariate model only included one potential factor and multivariate model was additional adjusted for Leaders’ birth place, national capital city, total population number, population density and nighttime light. *P* values were reported for both models. Missingness on individual variables < 10%, except for ACT used in treatment (23%, *n* = 49 principle subdivisions) and access to an insecticide-treated mosquito net (19%, *n* = 41 principle subdivisions)

## Discussion

### Interpretation

Few studies have described the subnational distribution of China’s DAH projects and assessed the determinants of project allocations. In this study, we found that China’s DAH projects was mainly distributed to sub-Saharan Africa, which is the region with the lowest development level and poor health condition [30]. However, these projects were allocated along the western and eastern coasts of the continent where the economic development level was comparatively higher than that of the remaining regions [31]. Inland Africa is also in need of health facilities and health care service (Additional file 2: Figure S2), but few DAH projects from China have been implemented in this region. Interestingly, we observed an inverse association between nighttime lights and CMT allocation in the multivariate adjusted model, suggesting that some of the CMTs were dispatched to less developed areas in Africa. The slight difference between the allocation of CMTs and health facilities (hospitals and anti-malaria centers) could be a result of different implementation agencies for these projects; CMTs are dispatched by the National Health Commission, whereas health facilities are constructed with support from the Ministry of Commerce.

Our study showed a significant association between national capital cities and the allocation of China’s DAH projects. Subnational allocation of DAH projects from other governments has not been studied. However, within African countries, subdivisions with a greater proportion of individuals living in urban areas and more health facilities were more likely to receive DAH [32], which fitted the typical characteristics of a capital city and supported our findings. This finding also suggested that allocation of China’s DAH projects followed an approach similar to that of other donors. Since DAH is one part of development assistance and also a diplomatic tool [33], allocating DAH projects in or around the national capital city might be an efficient approach to exert political and diplomatic influence. Meanwhile, the central governments of African countries can show their achievements in construction and health service delivery more obviously in national capital cities, which can also benefit more people.

Same as China’s overall development assistance [11], we observed a slightly increased probability of allocating CMTs in national leaders’ birth places. From the patronage politics view, African leaders sometimes used development assistance projects to favor people in their birth places [27], and CMTs were suitable “gifts” to improve health of the local population. Since the leaders probably invested other resources to their birth places, and consequently promoted the economic development of these places, the effect of leaders’ birth places was attenuated after adjusting for demographic and economic factors.

Generally, China did not allocate DAH projects according to the health-related indicators of African countries’ principle subdivisions, such as health care accessibility and maternal and child health. The significant associations we found in the univariate analyses were confounded by the demographic, political and economic indicators. Interestingly, hospitals were more likely to be allocated to principal subdivisions with higher rates of facility delivery and lower rates of under-five mortality, which were not the prioritized locations for DAH. Although the Chinese government claimed that China’s development assistance was based on a “recipient driven” mechanism [12], our study suggested that this “recipient driven” mechanism was more likely to be “recipient’s government elite driven” rather than “recipient’s local health needs driven”, which did not always align with each other. Therefore, the capacity of government agencies (authorities) for development cooperation in both China and Africa should be improved for the planning and implementation of DAH projects. In addition, coordination and negotiation between different departments and stakeholders on the donor and recipient sides are recommended to balance the political and health benefits of DAH projects. As China has recently launched the new International Development Cooperation Agency to reduce aid fragmentation, it is also the right timing to add the local health assessment procedure when implementing the new series of DAH projects in low-income countries.

Typically, access to ITN and ACT treatment coverage are associated with a high malaria risk and high *Plasmodium falciparum* malaria prevalence [34, 35], which indicate intermediate/high endemicity zones for intervention. However, we did not find an association between allocation of anti-malaria centers and these two indicators. This null association was a typical example of less involvement of health experts during the project implementation stage. At the initiation stage, the 30 anti-malaria centers were similar to a political promise announced by the Chinese government without prior assessment. During the implementation stage, Chinese medical companies and the Economic and Commercial Counsellors’ Offices in African countries (in the capital cities) were the main stakeholders. Malaria experts were only invited to train the local health care providers to use the equipment when the projects were almost completed [36].

### Strengths and limitations

The main strength of our study is the comprehensive dataset of China’s DAH projects, which enables in-depth analysis on aid flow and allocations at the sub-national level. Other strength includes abundant information on political, demographic, health and socioeconomic factors of the principle subdivisions.

The present study had some limitations. Although we tried to cover all of China’s DAH projects in the analyses, some of the projects may not have been reported to the public. However, since the number of analyzed projects was in concordance with China’s commitments in the Forums on China-Africa Cooperation [37] and we have official confirmation from the implementation agency, the under-reported projects should be too few to bias our results. As information on the funding flow of China’s individual DAH projects was not accessible, we were not able to assess the magnitude of DAH allocation. Nevertheless, the amount of funding for each aided hospital or each anti-malaria center was quite similar [17], and consequently, our results could partly suggest the funding flow of DAH. Additionally, because we collected information (both the DAH outcomes and explanatory variables) from different data sources, the quality and validity of several indicators have not been tested. Although demographic factors might be influenced by the data quality, the most significant finding of capital cities should not have been biased. In addition, analyses of health and social indicators were based on only half of the aided African countries, which might have reduced the power to determine associations. However, the majority of the associations in the univariate models were significant and the effect was attenuated after adjusting for confounders, indicating that the non-significant estimates in the multivariate models were the results of confounding adjustment rather than a power issue. Finally, our analysis did not cover some other potential determinants, such as subnational trade with China, ethnic group and coverage of public health facility, which need further investigation. These findings were also limited to China’s DAH projects during 2006–2015 and the determinants might change in the aforementioned projects after 2015.

## Conclusions

Majority of China’s DAH projects are allocated to the western and eastern coasts of Africa, although CMTs are also dispatched to northern Africa. The sub-national allocation of China’s DAH projects is strongly influenced by political and demographic factors. Implementation of China’s new DAH projects should target health and socioeconomic indicators, which may consequently improve the effectiveness and precision of China’s aid projects.

## Additional files

Additional file 1: Multilingual abstracts in the five official working languages of the United Nations. (PDF 688 kb)

Additional file 2: Location of the China’s development assistance for health projects in Africa 2006–2015. **Figure S1.** Decision making mechanism of China’s DAH projects. Abbreviations: CMT: China medical team; DAH: Development Assistance for Health; MOH: Ministry of Health (renamed as National Health and Family Planning Commission from 2013); IHECC: International Health Exchange & Cooperation Centre; MOFCOM: Ministry of Commerce; MOF: Ministry of Finance; MOFA: Ministry of Foreign Affairs. This figure only illustrated the decision making mechanism for China’s DAH projects during 2006–2015. After the launch of the new International Development Cooperation Agency, it might be changed. Other forms of China’s DAH are represented by hospital construction and anti-malaria centers. **Figure S2.** Health indicators in Africa at the subnational level. Shapes of the principal subdivisions and the health indicators were obtained from the Demographic and Health Surveys website (http://www.dhsprogram.com). **Table S1.** Descriptive of indicators used in the analyses. (PDF 1886 kb)

## Abbreviations

ACT: Artemisinin-based combination therapy
CMTs: China medical teams
DAH: Development assistance for health
DHS: Demographic and Health Surveys
GADM: Database of Global Administrative Areas
GDP: Gross domestic product
ISO: International Organization for Standardization
ITN: Insecticide-treated mosquito net
