# Supplementary material for: Determinants of China’s development assistance for health at the sub-national level of African countries (2006–2015)
Source: Infect Dis Poverty. 2018 Dec 19;7:128. doi: 10.1186/s40249-018-0510-8 (PMC6307275; doi:10.1186/s40249-018-0510-8)
Supplement: Supplementary file 1 — Multilingual abstracts in the five official working languages of the United Nations. (PDF 688 kb) [file 40249_2018_510_MOESM1_ESM.pdf]

Translation of the abstract into the five official working languages of the United Nations

العوامل المحددة للمساعدة الإنمائية في الصين للصحة على الصعيد دون الوطني للبلدان الأفريقية (2006-2015)

هاو مين يانغ ، ليو باي طويلة ، يان قوه

نبذة مختصرة

معلومات أساسية:على الرغم من الاهتمام المتزايد بالمساعدات الإنمائية االصيينية للصحة (DAH) في البلدان الأفريقية، إلا أنه ليست هناك معلومات كافية عن توزيع والعوامل المحددة لتخصيص المشروع داه في الصين بين التقسيمات الفرعية االرئيسية (مقاطعات & الدول) داخل البلدان الأفريقية.
أساليب : وضعنا خريطة لتوزيع المشاريع DAH في الصين في 670 من التقسيمات الرئيسية في 50 بلدا أفريقيا خلال عام 2006 \u20122015 باستخدام معلومات على شبكة الإنترنت. تم تحليل المؤشرات السياسية والديمغرافية والصحية والاجتماعية الاقتصادية لتخصيص DAH باستخدام نماذج الانحدار اللوجستي الشرطي. العاصمة الوطنية و اختير مكان ولادة القائد السياسي كمؤشر سياسي أساسي، وجاء اختيار المؤشرات الصحية وفقاً لمجالات مختلفة من مشاريع DAH.
نتائج: مشاريع داه في الصين (أساسا الصين الفرق الطبية [CMTs] ومستشفيات ومراكز لمكافحة الملاريا) خصصت معظمها لسواحل افريقيا الشرقية والغربية، على الرغم من إيفاد CMTs إلى شمال أفريقيا أيضًا. العواصم الوطنية ترتبط ارتباطًا وثيقًا بتخصيص مشاريع DAH في الصين (ف < 0.001). من المرجح أن تُخصص مراكز لمكافحة الملاريا للتقسيمات الفرعية الرئيسية ذات أكبر عدد من السكان (أو = 1.35)، وخصصت CMTs إلى التقسيمات الفرعية ذات الكثافة السكانية العالية (أو = 79.01). لم تحدد أي مؤشرات متعلقة بالصحة تؤثر على توزيع المشروع باستثناء معدل تسليم المرفق ومعدل وفيات الأطفال دون سن الخامسة ، والمرتبط بتخصيص المستشفى. وجدنا أيضا وجود علاقة بين تخصيص CMT واستخدام العلاج المركب القائم على مادة الأرتيميسينين في الأطفال.
الاستنتاجات: يتأثر تخصيص مشروعات DAH الصينية بشدة بالعوامل السياسية والديموغرافية. يجب أن يستهدف تنفيذ مشاريع DAH الجديدة في الصين المؤشرات الصحية والاجتماعية الاقتصادية ومقاييس التأثير في توسيع نطاق البرامج المخصصة والفعالة من حيث التكلفة في أفريقيا.

Translated from English version into Arabic by Kowthar Alasady, proofread by Nader Hassan
, through

中国的卫生发展援助在非洲各国省级行政区间分布的影响因素（2006-2015）

杨昊旻，刘培龙，郭岩

摘要

引言：虽然现有文献对中国在非洲的卫生发展援助已有较多研究，但尚未有研究涉及援助项目在非洲各国内省级行政区之间的分布特点及其影响因素。
方法：本研究在搜集网络媒体信息的基础上，描述了 2006-2015 年中国在 50 个非洲国家的 670 个省级行政区中卫生发展援助项目的分布情况。应用条件 logistic 回归，分析了影响援助项目分布的政治、社会、经济、人口和健康等多方面因素。其中，国家首都所在地和领导人出生地是研究政治因素的主要指标，而健康方面的指标则视卫生发展援助项目的领域方向而定。
结果：中国的卫生发展援助项目（主要含中国援非医疗队、援建医院和援建抗疟中心）大多分布于非洲的东部和西部海岸线，其中援非医疗队在北非地区亦有驻点。中国卫生发展援助项目的实施地点同非洲国家的首都所

在地显著相关（$P < 0.001$）。抗疟中心建设更有可能被安排到人口较多的省级行政区（$OR = 1.35$），而援非医疗队则较多地被派遣到人口密集的地区（$OR = 79.01$）。除了援建医院的分布同住院分娩率和 5 岁以下儿童死亡率相关、援非医疗队的分布同青蒿素复方疗法在儿童中的治疗率相关之外，其他的健康相关指标同援助项目的分布并无显著关联。
结论：中国对非洲的卫生发展援助在各国省级行政区间的分布很大程度上受到当地政治和人口因素的影响。中国新一轮卫生发展援助项目的分布应当参考受援国内各地的健康和社会经济等指标，从而在非洲开展精准而有效的援助项目。

Translated from English version into Chinese by Hao-min Yang

Déterminants de l'aide au développement chinoise dans le secteur de la santé à l'échelle infranationale dans les pays africains (2006-2015)

Hao-min Yang, Pei-long Liu, Yan Guo

RÉSUMÉ

Contexte: Malgré l'intérêt croissant pour l'aide au développement chinoise dans le secteur de la santé (DAH) dans les pays africains, il existe peu de données sur la répartition et les déterminants de l'attribution de projets DAH chinois dans les principales subdivisions africaines (provinces & états).
Méthodes: Nous avons dressé une carte de la répartition des projets DAH chinois dans 670 subdivisions principales de 50 pays africains, au cours de la période allant de 2006 à 2015, en exploitant des données tirées de l'Internet. Les indicateurs politiques, démographiques, sanitaires et socio-économiques utilisés pour l'attribution de DAH ont été analysés à l'aide de modèles de régression logistique conditionnelle. La capitale du pays et le lieu de naissance du dirigeant politique ont été retenus comme indicateurs politiques principaux, et les indicateurs sanitaires ont été retenus selon différents domaines de projet DAH.
Résultats: projets DAH chinois (en particulier les équipes médicales [CMT], les hôpitaux et les centres contre le paludisme chinois) s'adressaient principalement aux côtes ouest et est de l'Afrique, même si des CMT étaient également envoyées en Afrique du Nord. Les capitales de pays comptaient pour beaucoup dans l'attribution de projets DAH par la Chine ($P < 0,001$). Les centres contre le paludisme étaient plus souvent ouverts dans des subdivisions majeures ayant une population plus importante ($OR = 1,35$), et les CMT étaient affectées dans des subdivisions ayant une forte densité de population ($OR = 79,01$). Il n'a pas été retrouvé d'indicateurs sanitaires influençant l'attribution de projet, à l'exception du taux de naissance en centre médical et du taux de mortalité des moins de cinq ans, deux indicateurs associés à l'ouverture d'un hôpital. Nous avons également observé une association entre l'affectation d'une CMT et l'utilisation de polythérapies à base d'artémisinine (ACT) chez les enfants.
Conclusions: L'attribution des projets DAH chinois est fortement influencée par des facteurs politiques et démographiques. La mise en œuvre des nouveaux projets DAH chinois devrait cibler des indicateurs et des mesures d'impact sanitaires et socio-économiques en vue de renforcer en Afrique des programmes adaptés et efficaces par rapport à leur coût.

Translated from English version into French by Cendrine Strevens, proofread by Suzanne Assenat, through

Детерминанты помощи Китая по развитию здравоохранения на субнациональном уровне африканских стран (с 2006 по 2015 год)

Хао-Минь Ян (Hao-min Yang), Пэй-Лун Лю (Pei-long Liu), Янь Го (Yan Guo)

АННОТАЦИЯ

Справочная информация: Несмотря на растущий интерес к помощи, оказываемой Китаем в области развития здравоохранения (ПРЗ) в африканских странах, мало что известно о детерминантах и распределении ресурсов в рамках китайского проекта ПРЗ среди главных подразделений (провинций и штатов) в африканских странах.

Методы: Мы отобразили охват китайскими проектами ПРЗ по 670 основным подразделениям 50 африканских стран в период с 2006 по 2015 год с использованием веб-информации. С помощью моделей условной логистической регрессии были проанализированы политические, демографические, социально-экономические показатели, а также показатели здравоохранения для распределения ресурсов ПРЗ. Столица государства иместо рождения политического лидера были выбраны как основные политические показатели, а показатели в области здравоохранения были выбраны согласно различных областей действия проектов ПРЗ.

Результаты: Проекты Китая в области ПРЗ (главным образом, китайские медицинские бригады [КМБ], больницы и центры по борьбе с малярией) в основном были рассредоточены в прибрежных районах Западной и Восточной Африки, хотя КМБ были также направлены в Северную Африку. Связь столиц государств с распределением китайских проектов ПРЗ оказалась существенной ($P < 0,001$). Центры по борьбе с малярией чаще открывались в главных подразделениях с большей численностью населения ($OR = 1,35$), а КМБ распределялись по подразделениям с высокой плотностью населения ($OR = 79,01$). Не было выявлено никаких индикаторов, связанных со здоровьем, которые бы оказали влияние на распределение проектов, за исключением процента родоразрешений в медицинских учреждениях и смертности детей в возрасте до пяти лет, которые были связаны с распределением больниц. Кроме того, мы обнаружили связь между распределением КМБ и применением комбинированной терапии на основе артемизинина для детей.

Выводы: На распределение ресурсов проектов Китая в области ПРЗ существенно повлияли политические и демографические факторы. Осуществление новых проектов Китая в области ПРЗ должно быть направлено на социально-экономические показатели, показатели здравоохранения, а также показатели воздействия, чтобы активизировать в Африке адаптированные и экономически эффективные программы.

Translated from English version into Russian by Liudmila Tomanek, proofread by Olga Madiar, through

Determinantes de la asistencia de China al desarrollo en el ámbito sanitario a nivel subnacional de los países africanos (2006-2015)

Hao-min Yang, Pei-long Liu, Yan Guo

RESUMEN

Introducción: A pesar del creciente interés en la asistencia de China al desarrollo en el ámbito sanitario (DAH, por sus siglas en inglés) en los países de África, se conoce muy poco acerca de la distribución y los determinantes de las asignaciones del proyecto de DAH de China entre las subdivisiones principales (provincias y estados) en los países de África

Métodos: Mapeamos la distribución de los proyectos de DAH de China en 670 subdivisiones principales de 50 países africanos durante 2006–2015 utilizando información basada en la web. Los indicadores políticos, demográficos, sanitarios y socioeconómicos de asignación de DAH fueron analizados usando modelos de regresión logística condicional. La capital nacional y el lugar de nacimiento del líder político fueron elegidos como los principales indicadores políticos, y se seleccionaron los indicadores de salud según distintos ámbitos de los proyectos de DAH.

Resultados: Los proyectos de DAH de China (principalmente los equipos médicos de China [CMT], hospitales y centros contra la malaria) se asignaron en gran parte a las costas occidental y oriental de África, aunque también se enviaron CMT al norte de África. Se asociaron significativamente las ciudades capitales nacionales a la asignación de proyectos de DAH de China ($P < 0{,}001$). Lo más probable fue que se asignaran los centros contra la malaria a las subdivisiones principales con poblaciones más grandes (*IO*= 1,35), y se asignaron CMT a subdivisiones con altas densidades poblacionales (*IO* = 79,01). No se identificaron indicadores relacionados con la salud que afecten la asignación del proyecto, excepto la tasa de partos en centros de salud y la tasa de mortalidad de menores de cinco años, que se asociaron a la asignación de hospitales. También se encontró una asociación entre la asignación de CMT y el uso de tratamientos combinados a base de artemisinina en los niños.

Conclusiones: La asignación de proyectos de DAH de China se ve fuertemente afectada por factores políticos y demográficos. La implementación de los nuevos proyectos de DAH de China debería apuntar a indicadores de salud y socioeconómicos y métricas de impacto en la ampliación de programas personalizados y rentables en África.

Translated from English version into Spanish by Eugenia Cagni, proofread by Kate Pattison, through

TRANSLATORS
WITHOUT BORDERS
