# Supplementary material for: Determinants of China’s development assistance for health at the sub-national level of African countries (2006–2015)
Source: Infect Dis Poverty. 2018 Dec 19;7:128. doi: 10.1186/s40249-018-0510-8 (PMC6307275; doi:10.1186/s40249-018-0510-8)

Additional file 2. Location of the China's development assistance for health projects in Africa 2006-2015

A. Location of China Medical Teams

Country	Principle subdivision	ISO-3166 code	No. of team member
Angola	Luanda	AO-LUA	11
Burundi	Bubanza	BI-BB	13
Burundi	Bujumbura Mairie	BI-BM	7
Burundi	Gitega	BI-GI	9
Benin	Atakora	BJ-AK	13
Benin	Littoral	BJ-LI	3
Benin	Mono	BJ-MO	10
Botswana	Gaborone	BW-GA	22
Botswana	North-East District,Francistown	BW-NE	24
D.R. Congo	Kinshasa	CD-KN	18
Congo	Brazzaville	CG-BZV	24
Congo	Pointe-Noire	CG-16	9
Cameroon	Centre	CM-CE	30
Cameroon	North	CM-NO	15
Cape Verde	Praia	CV-PR	8
Djibouti	Arta	DJ-AR	8
Djibouti	Djibouti	DJ-DJ	6
Algeria	Ain Defla	DZ-44	missing
Algeria	Alger	DZ-16	missing
Algeria	Batna	DZ-05	missing
Algeria	Khenchela	DZ-40	8
Algeria	Mascara	DZ-29	missing
Algeria	Saida	DZ-20	11
Algeria	Sétif	DZ-19	missing
Algeria	Tiaret	DZ-14	missing
Eritrea	Maekel Region	ER-M	18
Ethiopia	Addis Ababa	ET-AA	15
Gabon	Estuaire	GA-1	3
Gabon	Haut-Ogooué	GA-2	14
Ghana	Greater Accra	GH-AA	11
Guinea	Conakry	GN-C	19
Equatorial Guinea	Bioko Norte	GQ-BN	17
Equatorial Guinea	Litoral	GQ-LI	10
Guinea-Bissau	Bissau	GW-BS	14
Guinea-Bissau	Cacheu	GW-CA	3
Comoros	Anjouan	KM-A	3
Comoros	Grande Comore	KM-G	7
Comoros	Mohéli	KM-M	2
Liberia	Montserrado	LR-MO	9
Lesotho	Leribe	LS-C	9
Morocco	Chaouia-ouardigha	MA-09	10
Morocco	Grand casablanca	MA-08	3
Morocco	Marrakech-Tensift-Al Haouz	MA-11	8
Morocco	Meknès-Tafilalet	MA-06	20
Morocco	Rabat-Salé-Zemmour-Zaer	MA-07	4
Morocco	Souss-Massa-Drâa	MA-13	8
Morocco	Tanger-Tétouan	MA-01	12

Morocco	Taza-Al Hoceima-Taounate	MA-03	12
Madagascar	Analamanga	MG-T	12
Madagascar	Atsimo-Andrefana	MG-F	7
Madagascar	Atsinana	MG-A	4
Madagascar	Sava	MG-D	7
Mali	Bamako	ML-BKO	31
Mauritania	Assaba	MR-03	8
Mauritania	Guidimak	MR-10	7
Mauritania	Nouakchott	MR-NKC	12
Malawi	Lilongwe	MW-LI	8
Malawi	Mzimba	MW-MZ	8
Mozambique	Maputo	MZ-L	14
Namibia	Khomas Region	NA-KH	4
Niger	Maradi	NE-4	17
Niger	Niamey	NE-8	11
Niger	Zinder	NE-7	9
Rwanda	Kigali	RW-01	16
Seychell	Greater Victoria	SC-18	6
Sudan	Blue Nil	SD-NB	14
Sudan	Khartoum	SD-KH	28
Sierra Leone	Western Area	SL-W	10
Senegal	Dakar	SN-DK	13
South Sudan	Central Equatorial	SS-EC	12
Chad	N'Djamena	TD-ND	12
Togo	Kara	TG-K	10
Togo	Maritime	TG-M	11
Tunisia	Jendouba	TN-32	13
Tunisia	Medenine	TN-82	11
Tunisia	Sidi Bou	TN-43	13
Tunisia	Tunis	TN-11	8
Tanzania	Dar es Salaam	TZ-02	11
Tanzania	Dodoma Region	TZ-03	6
Tanzania	Mara Region	TZ-13	4
Tanzania	Tabora Region	TZ-24	4
Tanzania	Zanzibar west	TZ-15	12
Tanzania	Pemba south	TZ-10	9
Uganda	Kampala	UG-102	8
Zambia	Copperbelt Ndola	ZM-08	9
Zambia	Lusaka	ZM-09	15
Zambia	Southern	ZM-07	4
Zimbabwe	Harare	ZW-HA	10

B. Location of China aided hospitals

Country	Principle subdivision	Name of hosptial	ISO-3166 code	Year
Togo	Maritime Region	Central Hospital in Lome	TG-M	2009
Guinea	Conakry	China-Guinea Friendship Hospital	GN-C	2010
Liberia	Nimba State	Tappita Hospital in Nimba State	LR-NI	2010
Mali	Bamako	Bamako General Hospital	ML-BKO	2010
Ghana	Greater Accra	Ghana General Hospital	GH-AA	2010
Benin	Borgou	Parakou Hospital	BJ-BO	2010
Mauritania	Nouakchott	China-Mauritania Friendship Hospital	MR-NKC	2010
Guinea-Bissau	Bissau	Military General Hospital of Guinea-Bissau	GW-BS	2010
Serra Leone	Western Area (Freetown)	Freedom Hospital	SL-W	2011
Cote-Ivoire	Fromager	Gagnoa Hospital	CI-18	2011
Burundi	Bubanza	Mbanda General Hospital	BI-BB	2011
Rwanda	Kigali	Masaka General Hospital	RW-01	2011
Tanzania	Dar es Salaam	Cardiac Surgery Center for Treatment and Training	TZ-02	2011
Seychelles	Anse Royale	Anse Royal Hospital	SC-05	2011
Djibouti	Arta	Arta Hospital	DJ-AR	2011
Kenya	Coast	Lucy Kibaki Hospital	KE-300	2011
Sudan	Blue Nile State	Ad-Damazin General Hospital in Blue Nile State	SD-NB	2013
Uganda	Central	China-Uganda Friendship Hospital, Naguru	UG-C	2011
Ethiopia	Addis Ababa	Tirunesh-Beijing Hospital	ET-AA	2011
Namibia	Oshikoto	Omuthiya General Hospital	NA-OT	2011
Zambia	Lusaka	Lusaka Central Hospital	ZM-09	2011
Nigeria	Federal Capital Territo	Nigeria State Employee’s Hospital	NG-FC	2012
Zimbabwe	Mashonaland East	Hospital in Marondera	ZW-ME	2012
Senegal	Dakar	Senegal Children Hospital	SN-DK	2012
Central Africa	Ombella-M'Poko	Central Africa Binbo hospital	CF-MP	2012
Cape Verde	Praia	Outpatient building and obstetrics building of Praia Central Hospital	CV-PR	2013
Comores	Nzwani	A hospital in Bambao, Anjouan	KM-A	2012
Kenya	Central	Gatundu Hospital Expending	KE-200	2013
Cameroon	Littoral	Duala Women & Children Hospital	CM-LT	2014
Congo	Kouilou	Mfilou Hospital	CG-5	2013
Angola	Luanda	Extension of Luanda General Hospital	AO-LUA	2014
South Sudan	El Buheyrat	Rumbek Hospital	SS-LK	2013
Mauritius	Plaines Wilhems	Operation Center and wards in Victoria Hospital	MU-PW	2013
Niger	Niamey	General Hospital in Niamey	NE-8	2013
Mauritania	Assaba	Kiffa health center	MR-03	2015

C. Location of China aided anti-malaria centers

Country	Principle subdivision	ISO-3166 code	Year
Liberia	Montserrado	LR-MO	2007
Sudan	Al Jazeera	SD-GZ	2009
Mozambique	Maputo	MZ-L	2009
Ethiopia	Oromia	ET-OR	2008
Cameroon	Centre	CM-CE	2009
Uganda	Central	UG-C	2008
Tanzania	Dar es Salaam	TZ-02	2009
Sierra Leone	Western	SL-W	2009
Zambia	Lusaka	ZM-09	2009
Madagascar	Antananarivo	MG-T	2009
Angola	Luanda	AO-LUA	2009
Burundi	Bujumbura Mairie	BI-BM	2008
Congo	Brazzaville	CG-BZV	2008
Equatorial-Guinea	Litoral	GQ-LI	2009
Comores	Mwali	KM-M	2010
Gabon	Estuaire	GA-1	2008
Niger	Niamey	NE-8	2012
Guinea-Bissau	Bissau	GW-BS	2008
Benin	Mono	BJ-MO	2008
Togo	Maritime	TG-M	2009
Ghana	Greater Accra	GH-AA	2009
Cote-Ivoire	Lagunes	CI-01	2009
Central Africa	Bangui	CF-BGF	2009
Chad	N'Djamena	TD-ND	2007
Guinea	Conarky	GN-C	2009
Mali	Bamako	ML-BKO	2009
Senegal	Dakar	SN-DK	2009
Kenya	Nairobi	KE-110	2009
Nigeria	Federal Capital Territory	NG-FC	2009
Rwanda	Kigali	RW-01	2011

Table S1. Descriptive of indicators used in the analyses.

Variable name	Medical teams		Hospitals		Anti-malaria centers	
	No	Yes	No	Yes	No	Yes
**Political characteristics**						
Birth places of national leaders						
No	434	50	463	21	463	21
Yes	59	20	68	11	70	9
Capital city of the country						
No	569	47	598	18	611	5
Yes	19	35	37	17	29	25
**Demographic and economic indicators**						
No. of population (million)	1.34	1.48	1.35	1.54	1.28	2.97
Population density (1000/km2)	0.16	1.33	0.26	1.02	0.22	2.09
Nighttime light (scale)	2.72	6.62	3.00	6.96	3.01	7.34
**Causes of Health facility accessibility problems**						
Distance (% of the population)	45.48	33.33	44.00	31.79	44.05	32.03
Finance (% of the population)	56.12	45.79	54.71	46.42	54.61	48.14
Transport (% of the population)	45.07	32.35	43.36	32.77	43.44	32.65
**Maternal and Child health**						
Facility delivery (% of live births in the previous 5 years)	47.80	60.83	48.48	69.98	48.57	68.97
Under 5 mortality rate(per thousand live births in previous 5 years)	140.02	109.63	136.69	104.50	136.49	108.41
Low birth weight (% of live births in previous 5 years)	11.06	11.55	11.27	9.78	11.09	11.79
**Malaria prevention and treatment**						
Access to an insecticide-treated mosquito net (% of the population)	10.49	15.60	11.03	13.20	10.94	14.88
ACT used in treatment(% of child with fever)	3.24	8.03	4.15	2.14	4.02	3.15
**Social factors**						
Male literacy rate (% of the population)	67.84	78.12	68.47	80.89	68.55	80.08
Female literacy rate (%of the population)	51.56	62.46	52.35	67.61	52.66	64.10
Male unemployment rate (% of the population)	24.81	23.62	24.56	25.27	24.68	23.86
Female unemployment rate (%of the population)	37.35	44.13	38.38	41.30	38.37	41.40

Figure S1

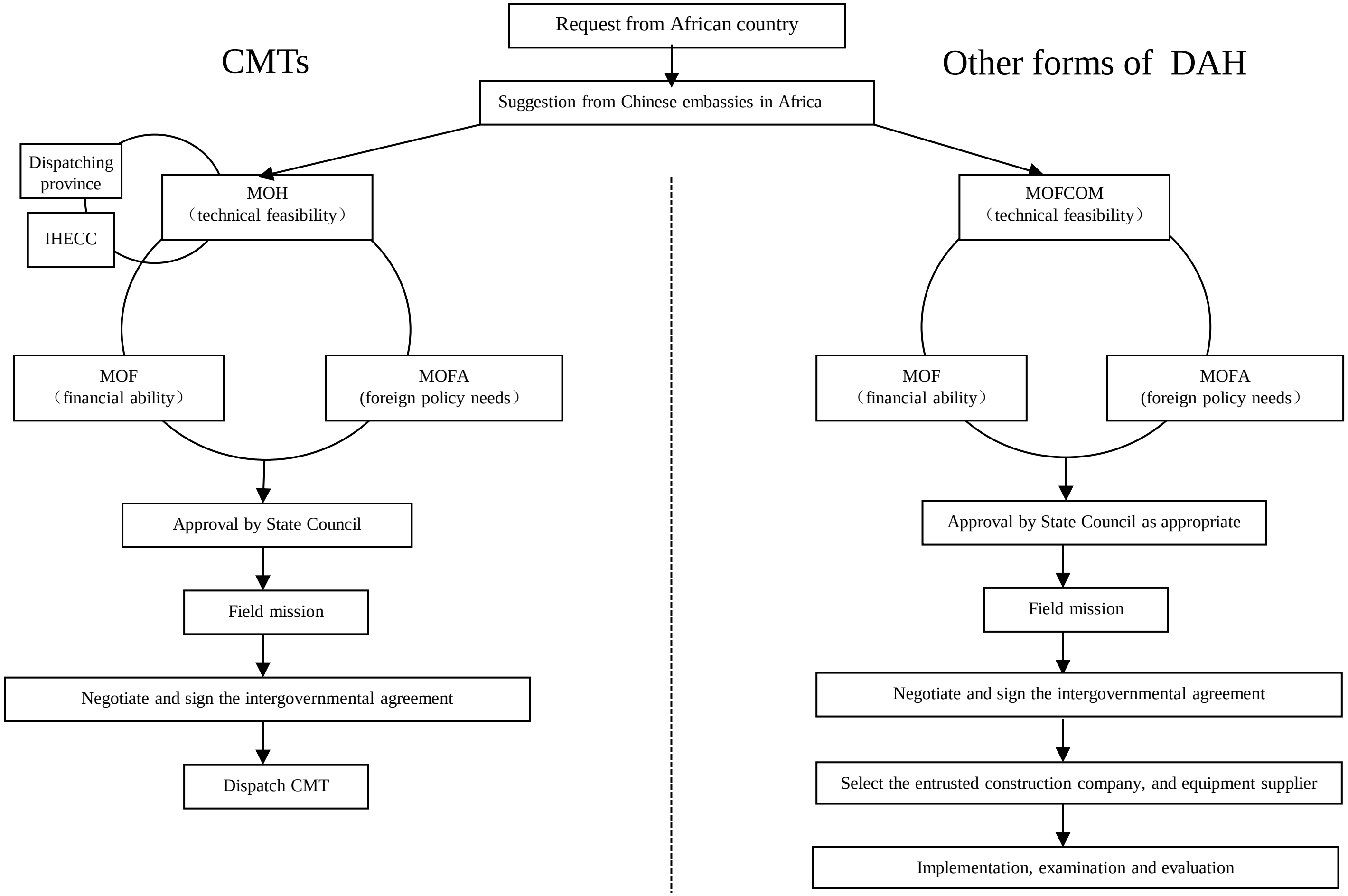
Request from African country
CMTs
Other forms of DAH
Suggestion from Chinese embassies in Africa
Dispatching province
IHECC
MOH （technical feasibility）
MOFCOM （technical feasibility）
MOF （financial ability）
MOFA (foreign policy needs）
MOF （financial ability）
MOFA (foreign policy needs）
Approval by State Council
Approval by State Council as appropriate
Field mission
Field mission
Negotiate and sign the intergovernmental agreement
Negotiate and sign the intergovernmental agreement
Dispatch CMT
Select the entrusted construction company, and equipment supplier
Implementation, examination and evaluation

Figure S2

Problem in accessing health care

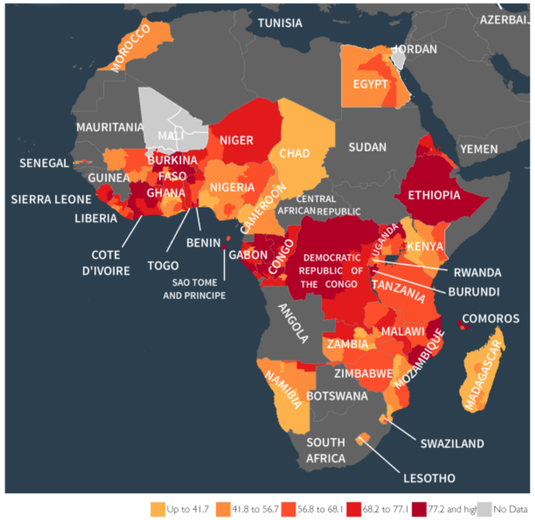

Facility based delivery

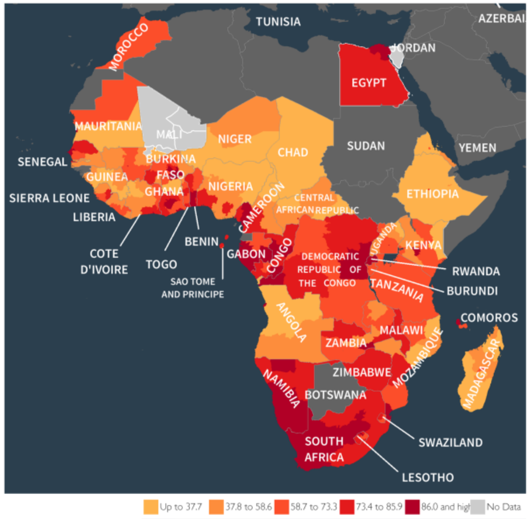

Under 5 mortality rate

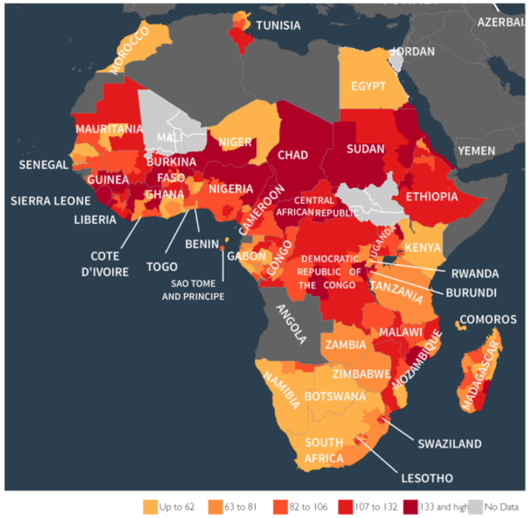

Rate of low birth weight

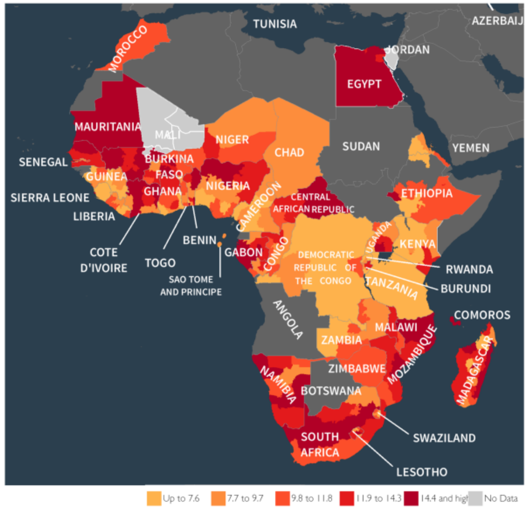

Access to insecticide-treated mosquito net

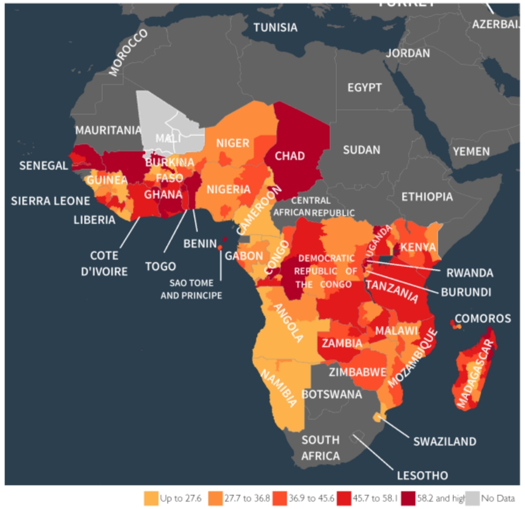

Treated with ACT

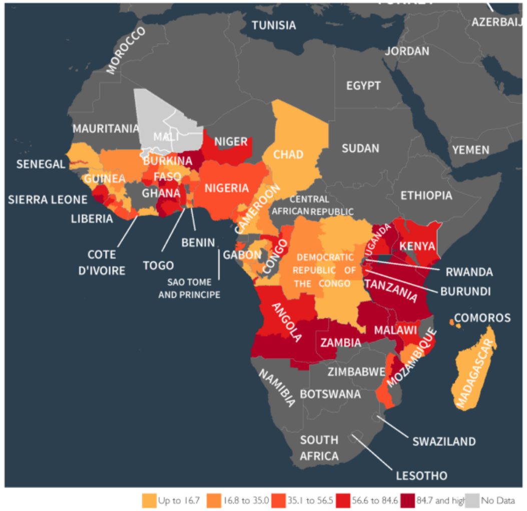

Supplement: Supplementary file 2 — Location of the China’s development assistance for health projects in Africa 2006–2015. Figure S1. Decision making mechanism of China’s DAH projects. Abbreviations: CMT: China medical team; DAH: Development Assistance for Health; MOH: Ministry of Health (renamed as National Health and Family Planning Commission from 2013); IHECC: International Health Exchange & Cooperation Centre; MOFCOM: Ministry of Commerce; MOF: Ministry of Finance; MOFA: Ministry of Foreign Affairs. This figure only illustrated the decision making mechanism for China’s DAH projects during 2006–2015. After the launch of the new International Development Cooperation Agency, it might be changed. Other forms of China’s DAH are represented by hospital construction and anti-malaria centers. Figure S2. Health indicators in Africa at the subnational level. Shapes of the principal subdivisions and the health indicators were obtained from the Demographic and Health Surveys website (http://www.dhsprogram.com). Table S1. Descriptive of indicators used in the analyses. (PDF 1886 kb) [file 40249_2018_510_MOESM2_ESM.pdf]
